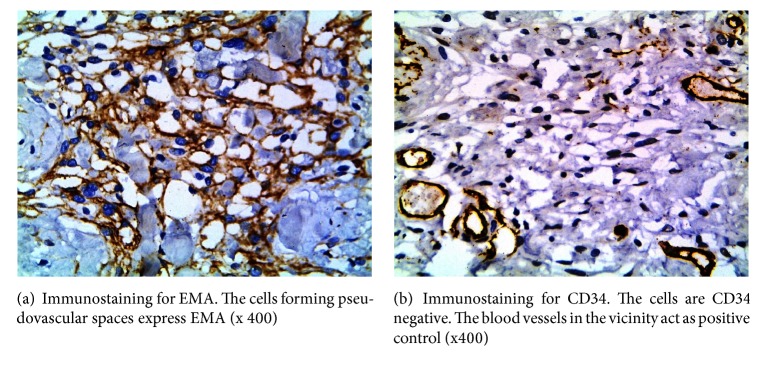# Rudimentary Meningocele of the Scalp: A Pitfall in the Diagnosis of Vascular Neoplasms

**DOI:** 10.1155/2018/4847286

**Published:** 2018-12-26

**Authors:** Subramaniam Ramkumar

**Affiliations:** Consultant in Pathology, Neuberg Anand Reference Laboratories, Neuberg Diagnostics Pvt. Ltd., Thombra Arcade, Elamakkara Road, Kaloor, Cochin-682017, India

## Abstract

We present a case of sequestrated meningocele in a 1-year-old girl, who presented with a 1x1cm occipital swelling since birth. CT brain revealed the soft tissue swelling to be extracranial. She underwent surgical excision of the specimen and the excised mass was sent for histopathological examination. The specimen consisted of skin and subcutaneous tissue measuring 2 x 1 x 1 cm. The entire tissue was paraffin processed. Multiple sections studied from the lesion showed an ill circumscribed locally infiltrative dermal lesion. The lesion was composed of whorled proliferations of meningothelial cells enclosing pseudovascular spaces. Immunohistochemically the lesion was positive for EMA, Desmin, and negative for endothelial markers. The present case was documented as a rare case of a rudimentary meningocele.

## 1. Introduction

Rudimentary meningoceles are also known as primary cutaneous meningioma or acoelic meningothelial hamartoma. They are rare lesions which are very common in the scalp region. A 1-year-old girl presented with a 1 x 1 cm occipital swelling, present since birth, with no associated neurological symptoms. CT brain revealed the soft tissue swelling to be extracranial with calcific specks. Per operatively, the swelling was located in the occipital skin with no intracranial connection. It was excised and sent for histopathological examination to our laboratory. The specimen consisted of skin and subcutaneous tissue measuring 2 x 1 x 1 cm. A nodular projection 1 cm across was present on the skin. Cut surface of the specimen was diffusely grey white and smooth with no distinct nodularity or cysts. The entire tissue was paraffin processed.

Multiple sections studied from the lesion showed an ill circumscribed locally infiltrative dermal lesion. The lesion was composed of whorled proliferations of meningothelial cells enclosing pseudovascular spaces. Immunohistochemically the lesion was positive for EMA, Desmin, and negative for endothelial markers. The present case was documented as a rare case of a rudimentary meningocele. Awareness in morphological assessment and judicious application of immunohistochemistry help in the recognition of rudimentary meningocele despite its angiomatoid and pseudoinfiltrative components.

## 2. Case Presentation

A one-year-old girl presented with a 1 x 1 cm occipital swelling, present since birth, with no associated neurological symptoms. CT brain revealed the soft tissue swelling to be extracranial with calcific specks. Operatively, the swelling was located in the occipital skin with no intracranial connection. It was excised and sent for histopathological examination.

The specimen consisted of skin and subcutaneous tissue measuring 2 x 1 x 1 cm. A nodular projection 1 cm across was present on the skin. Cut surface of the specimen was diffusely grey white and smooth with no distinct nodularity or cysts. The entire tissue was paraffin processed.

**Figure 1:**
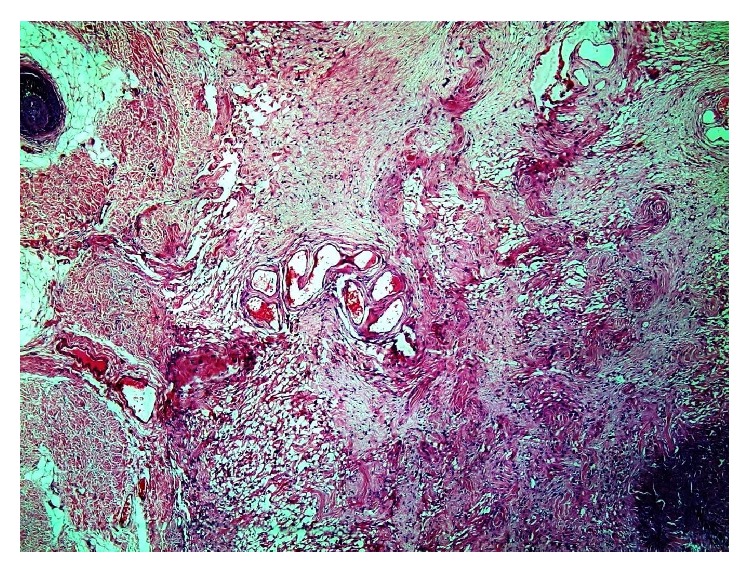
Photomicrograph shows an ill-defined lesion composed of haphazardly oriented collagen with lobules of fat, clusters of blood vessels and pseudovascular spaces (H and E x 100).

Haematoxylin–eosin stained sections showed the lesion, located in the deep dermis and subcutis, with ill-defined boundaries and consisting of haphazardly oriented collagen bands, lobules of fat, and clusters of blood vessels (Figure 1).

**Figure 2:**
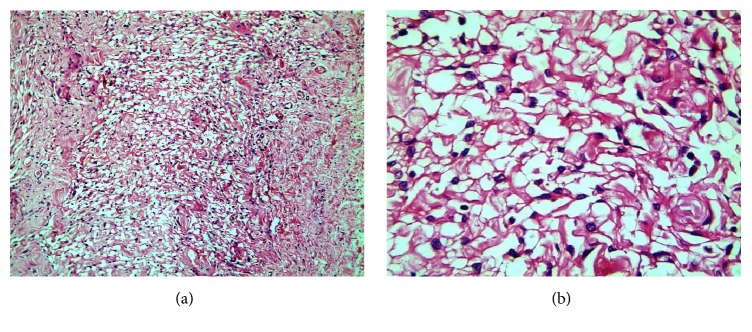
(a) (x100) and (b) (x400) view show round to spindle shaped nuclei protruding in to the pseudovascular spaces (H and E x 400).

The connective tissue was edematous at places and showed many plump fibroblastic cells. A striking feature was the presence of anastamosing spaces resembling vascular channels. These, along with the prominent clusters of larger blood vessels, suggested the possibility of an angiomatous neoplasm or hamartomatous lesion. (Figure 2)

**Figure 3:**
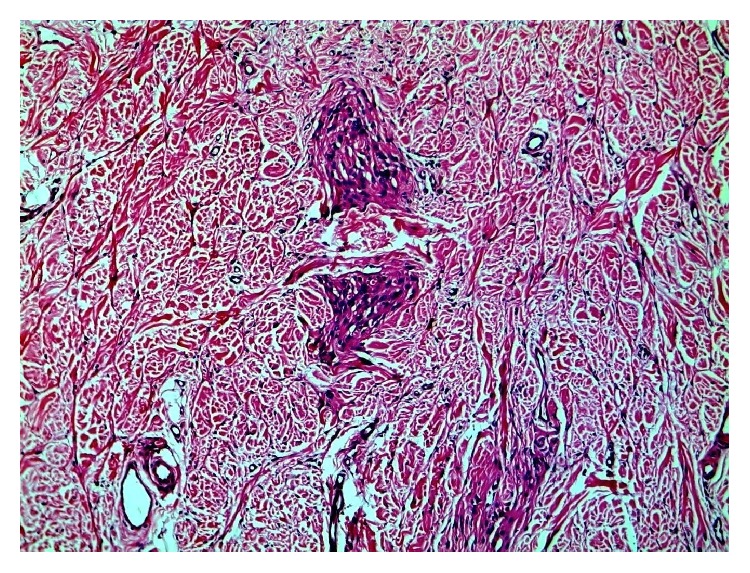
Clusters of cells insinuated between collagen fibers in a pseudoinfiltrative pattern (H and E x100).

**Figure 4:**
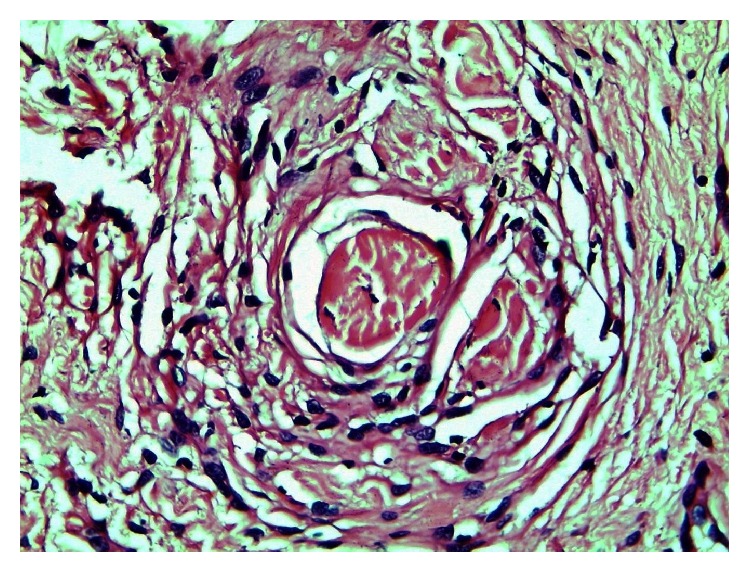
The figure shows a collagen body: concentric array of cells encircling collagen fibres (H and E x400).

Careful examination under higher magnification revealed a few irregular clusters of cells insinuated between collagen fibres and encircling collagen bands (Figures 3 and 4).

**Figure 5:**
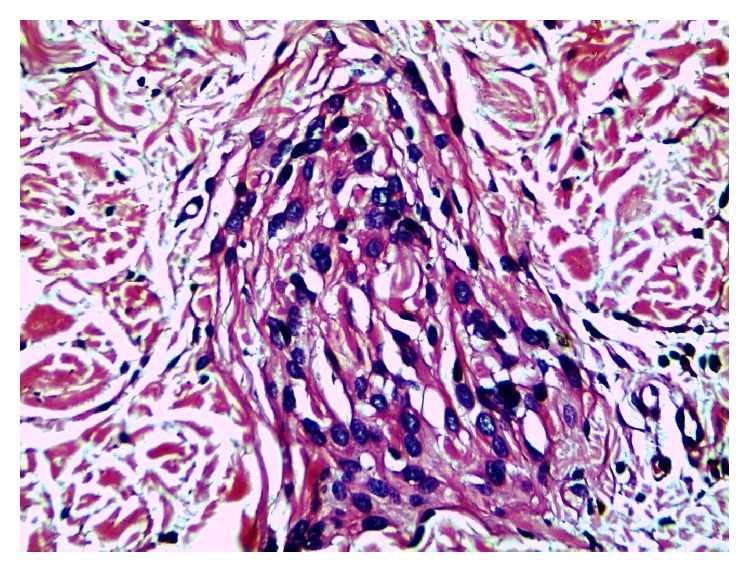
The cells show mild nuclear irregularity and hyperchromasia (H and E x400).

Some of these clusters showed mild nuclear irregularity and hyperchromasia (Figure 5).

Focal calcification was present along with a few histiocytes and giant cells around. Immunostaining was performed for meningothelial and endothelial markers. Cells lining the spaces and forming clusters

Strongly expressed vimentin and EMA. Endothelial markers (CD 34 and CD 31) gave negative results. (Figures 6(a) and 6(b)) [1].

A diagnosis of ectopic meningothelial hamartoma of the scalp was made.

## 3. Discussion

Rudimentary meningocele, also known as primary cutaneous meningioma or acoelic meningeal hamartoma, is an unusual developmental anomaly in which meningothelial elements are found in the subcutaneous tissue and deep dermis [1].

Lopez et al. in the year 1974 divided cutaneous meningiomas into three types, based on their study of twenty-five cases [2]. Type I, the congenital type, was usually located in the scalp or paravertebral region. In this, the authors described a subgroup which was ill-circumscribed, with scattered meningothelial cells and Psammoma bodies in streaks which they termed ‘acoelic meningioma,' to stress the close relationship of these lesions with meningoceles. Their description did not include atypical vessel like channels or abnormally arranged connective tissue elements.

A decade and a half later, in the year 1989, Sibley et al. described similar meningothelial lesions to Lopez et al. They highlighted that these lesions lacked the nodular and sheet-like growth patterns that typify meningiomas of the central nervous system and most primary ectopic meningiomas. They stressed the fact that these lesions appear closely related to meningoceles and should be viewed as developmental abnormalities rather than neoplasms. Hence they warranted that the term' rudimentary meningocele' was more appropriate for these lesions [1].

In the next year of 1990, Suster and Rosai reported five cases of “hamartoma of the scalp with ectopic meningothelial elements” [3]. The lesions were clinically benign. Microscopically, meningothelial cells in groups were admixed with haphazardly arranged connective tissue and blood vessels. A pseudoinfiltrative pattern and presence of anastamosing channels resembling blood vessels were highlighted in this report and the authors pointed out the importance of distinguishing these lesions from the more aggressive and neoplastic lesions like angiosarcoma. It was shown that the cells lining the spaces, though sometimes hyperchromatic, expressed vimentin, and EMA were not immunoreactive for endothelial markers {Factor  VIII  related  antigen  and  UlexEuropeus}.

Nevertheless, the case presented here would fit in to the Type I group of congenital meningothelial lesions described by Lopez et al. The clinical and pathological features of our case are also identical with those described by Suster and Rosai and Sibley et al. It reiterates the diagnostic pitfalls in classification terminology and respective histomorphology as pointed out by these authors.

Similar lesions have been described by others, under different names, such as sequestered meningocele and meningothelial hamartoma. [2]. Occurrence in older individuals [4] and association with nevus sebaceus and immature glandular components [5] have also been reported.

These lesions are believed to arise from meningothelial cells displaced due to defective closure of the neural tube or migration along cutaneous nerves [6–9]. Alternatively, it could represent a meningocele with obliterated intracranial connection. Data regarding specific genetic associations are lacking, but occurrence in siblings and in families spanning generations are on record [9].

The meningothelial cell or arachnoidal cell of the arachnoid membrane characteristically forms whorls with or without central calcification. Ultrastructure reveals intertwined cell processes held together by desmosomes. True to its epithelial phenotype, the meningothelial cell expresses epithelial membrane antigen {EMA} [3].

The morphological and immunohistochemical characteristics described above are reproduced in proliferative lesions of meningothelial cells, irrespective of location. While cellular whorls, psammoma/collagen bodies, and EMA expression help to confirm a meningothelial origin, angiomatoid channels present in some of these lesions pose a diagnostic problem. These spaces probably recapitulate the small subarachnoid spaces formed by delicate trabecular extensions of arachnoid into pia mater or result from detachment of desmosomes with breaking up of meningothelial cell processes. When in the classical location, as in intracranial microcystic meningiomas, the diagnosis is made easily, but difficulties could arise in ectopic locations.

Awareness in morphological assessment and judicious application of immunohistochemistry help in the recognition of rudimentary meningocele despite its angiomatoid and pseudoinfiltrative components. Prognosis following surgical excision is said to be very good.

## Figures and Tables

**Figure 6:**